# Serum Levels of Angiopoietin-Like Protein 2 and Obestatin in Iranian Women with Polycystic Ovary Syndrome and Normal Body Mass Index

**DOI:** 10.3390/jcm7070159

**Published:** 2018-06-22

**Authors:** Elham Rahmani, Samad Akbarzadeh, Ainaz Broomand, Fatemeh Torabi, Niloofar Motamed, Marzieh Zohrabi

**Affiliations:** 1 Department of Obstetrics and Gynecology, Faculty of Medicine, Bushehr University of Medical Sciences, Bushehr 7518759577, Iran; Ermrat1350@yahoo.com; 2 Department of Biochemistry, Faculty of Medicine, Bushehr University of Medical Sciences, Bushehr 7518759577, Iran; Smdakbarzadeh@yahoo.com; 3 Faculty of Medicine, Bushehr University of Medical Sciences, Bushehr 7518759577, Iran; AinazBroomand@yahoo.com (A.B.); Ft.torabi69@yahoo.com (F.T.); 4 Department of Community Medicine, Faculty of Medicine, Bushehr University of Medical Sciences, Bushehr 7518759577, Iran; motamedn@bpums.ac.ir; 5 The Persian Gulf Tropical Medicine Research Center, Bushehr University of Medical Sciences, Bushehr 7514947932, Iran

**Keywords:** polycystic ovary syndrome, insulin resistance, angiopoietin-like protein 2, obestatin, normal BMI

## Abstract

Background: Polycystic ovary syndrome (PCOS) is a common endocrine disease in women of reproduction age and a major cause of anovulatory infertility. Insulin resistance plays an important role in the development and durability of this disorder. ANGPTL2 is known as an inflammatory mediator derived from adipose tissue that links obesity to systemic insulin resistance, and obestatin has been identified as a hormone associated with insulin resistance that suppresses food reabsorption, inhibits gastric emptying and decreases weight gain. The aim of this study was to evaluate serum levels of ANGPTL2 and obestatin in PCOS women with normal body mass index (BMI). Methods: In this case-control study, 26 PCOS women based on the Rotterdam 2003 diagnostic criteria as the case group and 26 women with normal menstrual cycles as the control group were enrolled. Serum levels of ANGPTL2, obestatin, insulin and other hormone factors related with PCOS were measured by ELISA method and biochemical parameters were measured by an autoanalyzer. Data were analyzed by independent samples-*T* test, Chi Square, Correlation and a single sample Kolmogrov–Smirnov test using SPSS software, version 16. Results: There were no significant variations in the amount of ANGPTL2, obestatin, cholesterol, low-density lipoprotein-cholesterol, high-density lipoprotein, cholesterol, creatinine and dehydroepiandrosterone-sulfate between the two groups. There were significant increases in serum levels of fasting blood sugar (*p* = 0.01), insulin (*p* = 0.04), homeostasis model assessments of insulin resistance (*p* = 0.04), testosterone (*p* = 0.02), luteinizing hormone (*p* = 0.004), luteinizing hormone/follicle stimulating hormone (*p* = 0.006) and prolactin (*p* = 0.04) in case group compared to the control group. A significant positive correlation was observed between ANGPTL2 and insulin (*p* = 0.02), HOMA-IR (*p* = 0.01) and, on the other hand, a significant negative correlation was observed between obestatin and insulin (*p* = 0.01), HOMA-IR (*p* = 0.008) in PCOS group. Conclusions: In this study, no significant variations were observed in serum levels of ANGPTL2 and obestatin in PCOS women with normal BMI.

## 1. Introduction

Polycystic ovary syndrome (PCOS) is a common endocrine disease in women of reproduction age, which increases the incidence of infertility [1]. The prevalence of PCOS is 5–15% based on diagnostic criteria [2]. The Rotterdam 2003 proposed that the presence of at least two of the three criteria (oligo-/anovulation, hyperandrogenism and polycystic ovaries on ultrasonography) would be sufficient for PCOS diagnosis [3]. PCOS is a multi-factor disease that is involved with genetic and environmental factors and is associated with obesity, insulin resistance, glucose tolerance, dyslipidemia, and diabetes [4,5]. ANGPTL2 is a pro-inflammatory protein that is associated with chronic inflammatory diseases including diabetes, atherosclerosis and cancer that is extensively expressed in visceral adipose tissue [6]. ANGPTL2 has an important effect on angiogenesis, androgen biosynthesis, adipocyte function, and insulin resistance [7]. Studies show that circulating levels of ANGPTL2 in humans and mice are both dependent on insulin resistance, adiposity and inflammation and for this reason, ANGPTL2 is known as an inflammatory mediator derived from adipose tissue that links obesity to systemic insulin resistance [8,9,10], so that the removal of ANGPTL2 in obese mice resulted in a reduction in adipose tissue inflammation and systemic insulin resistance [8]. Studies show that the circulation level of ANGPTL2 decreases with weight loss, and this decrease in ANGPTL2 levels in obese diabetic patients was associated with a decrease in visceral fat tissue, so it can be stated that visceral fat is one of the primary sources of the circulation ANGPTL2 [11,12]. Studies have shown that the heart, kidneys, endothelial cells and macrophages can secrete large amounts of ANGPTL2 [13,14,15,16]. Therefore, ANGPTL2 is not necessarily secreted by adipose tissue, and different cells in the body can be a source of ANGPTL2. Previous studies in humans showed that due to the role of ANGPTL2 in inflammation, it is associated with developing type 2 diabetes so that inflammation in the Langerhans islets, fatty tissues, liver and muscles can promote insulin resistance and impairment of beta cell function [17,18].

Obestatin is a 23-amino acid peptide hormone that is produced by the post-translational modification of ghrelin precursor peptide (preproghrelin), which is mainly released from the stomach [19]. A lower level of obestatin is secreted in the pancreatic islets and other tissues, so that obestatin is secreted from ε-cells of the pancreas, while the ghrelin secretes from α, β and ε-cells of the pancreas [20]. Previous studies have shown that the effects of obestatin on appetite, food intake and gastric secretions is opposite to ghrelin [21,22]. Obestatin has been identified as a hormone associated with insulin resistance that suppresses food reabsorption, inhibits gastric emptying and decreases weight gain [23,24]. However, the true role of obestatin in the mechanism of obesity has not been fully determined yet; in some animal studies, it was found that obestatin unlike ghrelin may provide a new target for the control of obesity [25,26]. In a study on obese children, there was a significant positive correlation between obestatin and BMI [27] but in another study, serum levels of obestatin showed a significant decrease in Egyptian obese PCOS patients compared to non-obese PCOS patient and control group [28].

Because ANGPTL2 and obestatin were reported to be associated with insulin resistance, diabetes and obesity and due to the role of ANGPTL2 in inflammation, we hypothesized that they may also play a role in underlying pathogenic mechanisms which leads to PCOS. Since different cells in the body can be a source of ANGPTL2 and these factors have been studied in obese people so far, we have decided to evaluate serum levels of ANGPTL2 and obestatin in Iranian women with PCOS and normal BMI. 

## 2. Materials and Methods

### 2.1. Search Design

This research is a case-control study that included 26 Iranian women with PCOS based on the Rotterdam 2003 diagnostic criteria as the case group and 26 healthy Iranian women with normal menstruation periods referred to Abolfazle Clinic, Bushehr, Iran from April 2015 to October 2016 were selected as the control group.

### 2.2. Inclusion and Exclusion Criteria

In the case group, PCOS women based on the Rotterdam 2003 diagnostic criteria had at least two (hyperandrogenism clinical and showing up a polycystic ovary on ultrasound) of the three criteria [3] and healthy women were selected from women with normal menstruation periods and normal pelvic ultrasound, without acne, hirsutism and infertility.

Both groups matched in body mass index (mean BMI < 25 kg/m^2^) and age < 40 years. Participants did not have diseases such as thyroid, tumors, cardiovascular, diabetes, hypertension and renal disorders, and they should not have taken at least for 3 months contraceptive drugs such as: glucocorticoids, fertility, anti-diabetes, anti-obesity, anti-hypertension, anti-estrogenic and androgenic.

### 2.3. Sampling and Measurement of Parameters

In this study, PCOS women with delayed amenorrhagia for more than 6 months were excluded from the study. In PCOS and healthy women after menstruation, blood samples were obtained in the follicular phase (first to fourth day of menstruation) after 12 h of overnight fasting between the hours of 8:00–9:00 am. Serum was obtained after centrifugation (1000× *g* for 15 min at 4 °C) and then frozen at −80 °C. Anthropometric parameters including age, weight, height, body mass index and waist to hip ratio were measured using questionnaires, scales and meters. Biochemical parameters including fasting blood sugar (FBS), Cholesterol (Chol), Triglyceride (TG) and Creatinin (Cr) were measured with reliable enzymatic techniques (Pars Azmoon-Co., Tehran, Iran) by autoanalyser Selectra E (Vital Scientific N.V, Holliston, The Netherlands). The HDL-C levels were measured after precipitation with magnesium chloride by enzymatic techniques (Pars Azmoon-Co., Tehran, Iran) and the LDL-C levels were calculated by using the Friedewald formula [29]. The serum levels of Testosterone, Dehydroepiandrosterone-sulfate (DHEAS), Prolactin, LH, FSH (Pars Azmoon-Co., Tehran, Iran) and insulin, ANGPTL2, obestatin (Crystal Day, Shanghai, China) were measured by ELISA technique (Dynex Technologies 2cxb3510, Chantilly, VA, USA). Homeostasis model assessments of insulin resistance (HOMA-IR) was calculated by using the formula [30,31].

$$\mathrm{HOMA}.\mathrm{IR}=\frac{\mathrm{Fasting}\;\mathrm{Insulin}\left(µ\frac{\mathrm{Iu}}{\mathrm{mL}}\right)\times \mathrm{Fasting}\;\mathrm{Glucose}\left(\frac{\mathrm{mg}}{\mathrm{dL}}\right)}{405}$$

### 2.4. Ethical Consideration

The research was approved by the Ethics Committee of Bushehr University of Medical Sciences, (Code Number “iR.bpums.ac.rec.1394.100”) and in this study all participants reported their satisfaction.

### 2.5. Statistical Analysis

Statistical analysis of the data was performed using SPSS software version 16.0 (SPSS, Inc., Chicago, IL, USA). Independent samples-*T* test, Chi Squar and Correlation were used to compare the quantitative variables between the two groups and all results were expressed as mean ± SD. The single sample Kolmogrov–Smirnov test was used to estimate the variables’ distribution characteristics. Differences at the level of *p* < 0.05 were considered statistically significant. 

## 3. Results

In our study, there were no significant differences in demographic characteristics such as weight, height, age, BMI and WHR between the case and control groups (Table 1). A significant increase was observed in parameters involved in diabetes such as FBS (*p* = 0.01), insulin (*p* = 0.04) and HOMA-IR (*p* = 0.04) in the case group compared to the control group. There is a significant increase in the hormonal amount of LH (*p* = 0.004), LH/FSH (*p* = 0.006), Testosterone (*p* = 0.02), Prolactin (*p* = 0.04), in the PCOS group compared to the control group (Table 2). There were no significant differences in serum levels of Obestatin, ANGPTL2, Chol, HDL-C, LDL-C, TG, DHEAS, FSH and Cr in PCOS women compared to the control group (Table 2). A significant positive correlation was observed between ANGPTL2 and insulin (*p* = 0.02), HOMA-IR (*p* = 0.01) and on the other hand a significant negative correlation was observed between obestatin and insulin (*p* = 0.01), HOMA-IR (*p* = 0.008) in PCOS group (Table 3) and this correlation in control group was also observed but no significant correlation was observed between ANGPTL2 and obestatin with BMI in PCOS and control group. 

## 4. Discussion

In this study, there was no significant difference in serum levels of ANGPTL2 in PCOS women with normal BMI compared to the control group and there was also a significant positive correlation between ANGPTL2 and insulin, HOMA-IR. Sasaki Y and his colleagues stated that high expression of ANGPTL2 induced inflammation in adipose tissue and deteriorated insulin sensitivity [32]. On the other hands, Tabata M and his colleagues reported that in mice with defection in ANGPTL2, adipose tissue inflammation and insulin resistance improved [8]. Previous studies showed that the concentration of ANGPTL2 is regulated by the levels of obesity, so that levels of this factor decreased with weight loss and are significantly correlated with systemic insulin resistance and inflammation [11,33], also in a study conducted by Oike Y and his colleagues reported that the circulation levels of ANGPTL2 decreases in parallel with the reduction of adipose tissue in obese diabetic patients treated with pioglitazone, a drug that reduces adipose tissue and suppresses inflammation and increases insulin sensitivity [34]. Muramoto A and his colleagues stated that after a change in life style for a period of 3 months which continued for 6 months, a rapid decline was observed in ANGPTL2 levels in obese individuals that showed 2% or more weight loss [35]. Thorin-Trescases and colleagues reported in patients with acute coronary syndrome after 3 months of physical training, which the plasma levels of ANGPTL2 decreased to 26%, while body mass, lean and fat mass, waist circumference, and BMI were not affected by the exercise training program [36]. Also, in the study of Piche, M.E and colleagues found that after a bariatric surgery, a relative decrease (0% at 6 months, 18% at 1 year) in ANGPTL2 levels did not reflect a drastic decrease in weight (27% at 6 months, 37% at 1 year) and the two studies suggested that the decrease in ANGPTL2 levels after bariatric surgery or physical training is not a marker of adiposity [37], and it is stated that this factor also secreted by other cells such as the heart, kidneys, endothelial cells and macrophages [13,14,15,16].

In our study, there was no significant difference in the serum levels of obestatin in PCOS women compared to the control group, and on the other hand, a significant negative correlation was observed between obestatin and insulin, HOMA-IR, so that in the study of Abd El-Fattah AI and his colleagues serum levels of obestatin showed a significant decrease in Egyptian obese PCOS patients compared to non-obese PCOS patient and control group. There was a significant negative correlation between serum levels of obestatin and BMI, WHR, insulin, HOMAIR and it has been suggested that an increase in the rate of obesity in PCOS women may be attributed to lower obestatin levels in these individuals [28]. The reduction in the number of G protein coupled orphan receptors (GPR39) as obestatin receptor in diabetic patients confirms this statement [38] and in our study, a negative correlation was observed between obestatin and BMI in PCOS women with normal BMI but this was not significant. On the other hand, Razzaghy-Azar M and his colleagues reported that plasma levels of obestatin in obese children in fasting conditions were higher than the control group and found a significant positive relationship between obestatin and BMI [27]. Therefore, due to the opposite results, more studies are needed in order to determine the role of obestatin in weight control. 

Our results showed a significant increase in serum levels of insulin, HOMA-IR, FBS and other hormones, in accordance with these results (such as those of Gupta N and his colleagues) which reported that in all metabolic parameters including FBS, there was a significant difference between non-obese PCOS and control. The prevalence of IR in non-obese PCOS group was higher than the control group [39]. Also, Akbarzadeh S and his colleagues reported that there were significant increases in serum levels of insulin and HOMA-IR in the PCOS group compared to the control group; they also reported that in PCOS women with normal BMI, there is no significant difference in the levels of omentin and vaspin that are associated with insulin resistance [40].

## 5. Conclusions

In this study, although factors associated with the pathogenesis of PCOS including insulin resistance and HOMA-IR with ANGPTL2 and obestatin showed significant correlations, there was no significant difference in the serum levels of these two factors in PCOS women with normal BMI compared to the control group. Therefore, due to the different pathological effects of ANGPTL2 in the accumulation of inflammatory factors and induction of insulin resistance, it is also necessary to study this factor in obese PCOS women, but since obestatin, in our study and previous studies, showed significant correlations with the factors involved in the pathogenesis of PCOS, more studies are needed to clarify the pathological mechanisms associated with PCOS.

## Figures and Tables

**Table 1:** Demographic characteristics of case and control groups (Means ± SD).

Characteristic	Control Group	Case Group	*p* Value
Age (years)	28.91 ± 8.1	25.85 ± 5.90	0.12
Weight (kg)	62.47 ± 15.18	66.74 ± 12.41	0.28
Height (cm)	161.17 ± 4.71	159.52 ± 6.37	0.3
BMI (kg/m^2^)	24.02 ± 5.60	24.91± 3.63	0.4
WHR	0.8 ± 0.07	0.82 ± 0.06	0.27

Data presented as mean ± SD. The comparison was made using independent samples *t*-test, Chi square test. BMI: body mass index; WHR: waist–hip ratio.

**Table 2:** Serum concentrations of variables in case and control groups.

Characteristic	Control Group	Case Group	*p* Value
Obestatin (ng/mL)	16.55 ± 10.07	17.72 ± 10.56	0.69
ANGPTL2 (ng/mL)	143.69 ± 125.79	144.52 ± 131.84	0.98
Insulin (μIU/mL)	8.76 ± 6.63	16.42 ± 10.01	0.04
HOMA-IR	2.06 ± 2.04	4.11 ± 3.29	0.04
FBS (mg/dL)	91.29 ± 8.53	98.71 ± 11.44	0.01
Triglyceride (mg/dL)	86.79 ± 50.23	107.04 ± 45.03	0.13
Cholesterol (mg/dL)	167.83 ± 29.18	175.07 ± 23.32	0.3
HDL-C (mg/dL)	49.25 ± 12.59	47.92 ± 10.08	0.67
LDL-C (mg/dL)	100.96 ± 26.55	99.60 ± 25.72	0.62
LH (mIu/mL)	5.54 ± 2.84	9.46 ± 5.6	0.004
FSH (mIu/mL)	6.28 ± 2.13	5.24 ± 2.35	0.1
LH/FSH	1.06 ± 0.84	2.27 ± 1.92	0.006
Testosterone (ng/mL)	0.71 ± 0.26	0.99 ± 0.52	0.02
DHEAS (µg/dL)	1.83 ± 0.78	1.99 ± 0.87	0.4
Prolactin (ng/mL)	15.00 ± 8.84	21.23 ± 11.94	0.04
Cr (mg/dL)	0.80 ± 0.16	0.81 ± 0.13	0.7

Data presented as mean ± SD. Independent samples *t*-test, Chi square test, single sample Kolmogrov-Smirnov test. ANGPTL2: Angiopoietin-like protein 2. HOMA-IR: Homeostasis model assessments of insulin resistance; FBS: Fasting blood sugar; HDL-C: High-density lipoprotein cholesterol; LDL-C: Low-density lipoprotein cholesterol; LH: Luteinizing hormone; FSH: Follicle stimulation hormone; DHEAS: Dehydroepiandrosterone-sufate; Cr: Creatinine.

**Table 3:** The correlation between ANGPTL2 and obestatin with other variables in case and control groups.

		Correlation	BMI	Insulin	HOMA.IR
Case group	ANGPTL2 (ng/mL)	Pearson correlation	0.03	0.45	0.46
		*p*-value	0.86	0.02	0.01
	Obestatin (ng/mL)	Pearson correlation	−0.07	−0.49	−0.51
		*p*-value	0.74	0.01	0.008
Control group	ANGPTL2 (ng/mL)	Pearson correlation	0.37	0.39	0.40
		*p*-value	0.06	0.04	0.04
	Obestatin (ng/mL)	Pearson correlation	−0.30	−0.42	−0.39
		*p*-value	0.08	0.03	0.04
